# Synergistic Impact of Bioactive Byproduct Extract Leads to Anti-*Fusarium* and Anti-Mycotoxin Secretion

**DOI:** 10.3390/jof8010030

**Published:** 2021-12-29

**Authors:** Ahmed Noah Badr, Lukasz Stepien, Kinga Drzewiecka, Salman S. Alharthi, Khaled Selim, Adel Gabr Abdel-Razek

**Affiliations:** 1 Food Toxicology and Contaminants Department, National Research Centre, Cairo 12622, Egypt; 2 Pathogen Genetics and Plant Resistance Department, Institute of Plant Genetics, Polish Academy of Sciences, 60-479 Poznań, Poland; lste@igr.poznan.pl; 3 Chemistry Department, Poznan University of Life Science, 60-637 Poznań, Poland; kinga.drzewiecka@pracownik.up.poznan.pl; 4 Chemistry Department, College of Science, Taif University, P.O. Box 11099, Taif 21944, Saudi Arabia; s.a.alharthi@tu.edu.sa; 5 Food Science and Technology Department, Faculty of Agriculture, Fayoum University, Faiyum 63514, Egypt; kas00@fayoum.edu.eg; 6 Fats and Oils Department, National Research Centre, Cairo 12622, Egypt; adelgabr2@gmail.com

**Keywords:** antioxidant activity, anti-*Fusarium*, byproduct extract, deoxynivalenol, flavonoids, limonine, phenolic content, mycotoxin reduction, toxigenic fungi, zearalenone

## Abstract

Fruit byproducts are considered a high source of bioactive molecules, which possess antioxidant activities. These antioxidants play principal functions in mycotoxin reduction. This study aimed to evaluate crude mandarin byproduct extract for its chemical interaction with fungal growth and suppression of mycotoxin production, and to illustrate whether the impact was regarding individual molecules or a synergistic antioxidation process. Extract contents were analyzed for their phenolic, flavonoids, and antioxidant activity. The fatty acid composition and volatile components were determined using the GC apparatus. The influence of the extract evaluated versus the standard phenolics of trans-ferulic and hesperidin were evaluated. The liposome technique was applied to prevent the antioxidant properties of the bioactive extract. The anti-mycotoxigenic effects of the liposomal and non-liposomal extract were determined in fungal media against the standard phenolics. The results manifested ferulic (235.54 ± 3.34 mg/100 g) and hesperidin (492.11 ± 1.15 mg/100 g) as high phenolics in the extract. Limonene was the main volatile (67.54 ± 1.74%), as well antioxidant activities determined in considerable values. The crude extract recorded efficiency as an anti-*Fusarium* agent, but less than the standard hesperidin applied in fungal media. The bioactive extract recorded possessed a reduction influence on mycotoxin production. The impact may be joining with its fungal inhibition or its component activity with the active groups on the mycotoxin molecule. The formation of liposomal extract enhanced its efficacy in mycotoxin reduction. This enhancement may illustrate its protective properties for antioxidant components of the bioactive extract.

## 1. Introduction

Commonly, plant extracts are known as a wealthy source of minor components and nutrients. These nutrients possess a biological function in the metabolic pathways of living organisms [1]. The quantities of nutrients and minor components in the extracts are varied according to the conditions and the environment where the plants are grown [2], but dominant components that are distinguishing of each plant. The minor components and nutrients, which are knowing as bioactive components, could be playing significant functions in the biological systems [3]. These components, mainly acting as antioxidants, protect the tissues against the free radicals, and support the defense system. The components of plant extract that mainly possess this potency to act as antioxidant are the polyphenols.

Phenolic compounds are molecules that exist in natural extracts and have a biological impact [4]. Normally, phenolic compounds include phenolic acids and flavonoids, which are distinguished by the presence of aromatic rings with one or more hydroxyl groups. Flavonoids are the most abundant class of naturally occurring phenolic chemicals, found in plant components in the form of glycosides, aglycones, and methylated derivatives [5]. Reducers, free radical scavengers, and singlet oxygen quenchers all seem to be functions of phenolics and flavonoids [4]. Moreover, flavonoids and phenolic acids have vital functions in the treatment of various cancer types and other human illnesses [6,7]. Phenolics are readily absorbed through the wall of cells, similar to the epithelial cells of the gastrointestinal tract. These compounds have beneficial functions because of their potential role as antioxidants, which have a preventing role against cellular damage caused by free-radical oxidation [8,9,10].

Frequently, the living cell becomes damaged when exposed to free radicals. These free radicals could be produced intracellularly or associated with external stimuli, predominately chemical compounds [11]. Fungi are organisms that behave differently if they are exposed to inappropriate conditions in their environmental growth. Toxin-producing fungi, belonging to these species, are characterized by their ability to produce secondary metabolites called mycotoxins [12,13]. Two significant factors are related to mycotoxin production. Firstly, the ability of fungi to produce mycotoxins is often related to the suffering of the fungal cells from oxidative stress, which is known to force the cells to save their food as mycotoxin compounds. The second factor is that these mycotoxins, especially aflatoxins, can serve as free radicals and cause oxidative stress on living cells [13].

Previously, several investigations have pointed out the plant extracts’ functionality in mycotoxin-secretion reduction [14,15,16,17]. Nonpolar extracts of the plant materials that included non-traditional types of oils, tocopherol, tocotrienol, and sterol contents were counted to have a positive impact on mycotoxin-secretion reduction in liquid media [18,19]. The polar extracts that possess valuable contents of polyphenols, flavonoids, and antioxidants also limited the toxin amount secreted in fungal-growth media [20,21,22]. Moreover, it was referred to phenolic compounds as bioactive components with a considerable potency in mycotoxin reduction of fungal-growth media [13]. The anti-*Fusarium* effect of natural extracts was investigated before with recommendation for special extraction systems [23,24]. This could be a kick-off point for the present investigation.

To illustrate the reduction happened for mycotoxin production by means of the extracts existent in fungal media, it is significant to know whether this has occurred via an individual phenolic compound impact or via the synergism between the extract components. The present study aimed to test the influence of crude extract of mandarin byproducts comparing with two of its phenolic compounds (*t*-ferulic and hesperidin) for their antifungal activity against mycotoxigenic strains. The higher results recorded for the crude extract may be considered evidence about the synergistic effect between its components. Byproduct extract was evaluated in two forms, crude extract and liposomal extract. The impact was estimated in liquid fungal-growth media, as well as using a simulated experiment of food products. 

## 2. Materials and Methods

### 2.1. Chemicals, Microorganisms and Evaluated Material

The standard chemicals of the Supelco™ were used in our experiment, which comprised 6-Hydroxy-2, 5, 7, 8-tetramethylchromane-2-carboxylic acid (Trolox™) (Bellefonte, PA, USA). Where applicable all standards were purchased from Sigma-Aldrich Chemical Co. (St. Louis, MO, USA) included 2,2-diphenyl-1-picrylhydrazyl radical (DPPH), 2, 2′-Azino-bis(3-ethylbenzothiazoline-6-sulfonic acid) diammonium salt (ABTS™), trans ferulic acid, hesperidin, the phenol reagent of Folin & Ciocalteu’s phenol reagent, zearalenone (ZEA), ochratoxin A (OCA), and deoxynivalenol (DON).

The antifungal assay was conducted against toxigenic fungi as *Fusarium graminarum* KF841; *Aspergillusniger* ITEM 3856; *Penicilliumchrysogenum* ATCC 10106; *Penicilliumverrucosum* NRRL 965, *Fusariumculmorum* KF191; *Fusariumculmorum* KF846; *Fusariumoxysporum* ITEM 12591; *Fusariumverticillioides* FM19; *Fusariumculmorum* P846. These strains were isolated and identified in the plant pathology laboratory of the Polish Academy of Science’s Institute of Plant Genetics in Poznan, Poland, as these strains were referenced in a previous work [23]. The strains of *Fusarium* were reported by a capacity of producing the ZEA and the DON toxins, the strains of *A. niger* and *P. verrucosum* are examined to produce ochratoxin A (OCA) and ochratoxin B (OCB), and these results were a part of preliminary examination [23]. For the activation step, fungi were grown on malt extract media followed by a Czapek-Dox broth medium.

The fresh byproducts of mandarin (*Citrus sinensis*) fruits (consists of stalk, small leaves attached, and fruit peels) were collected from an ideal farm of citrus production located in Valley of the Angel, Ismailia governorate, Suez Canal area, Egypt. The requested numbers of mandarin byproducts were utilized immediately for further analysis.

### 2.2. Byproducts Extraction

Regard the previous extraction research applied to citrus products, a combination of microwave and ultrasonic extraction was utilized to achieve the effectiveness toward gaining the majority content of minor and bioactive components. The results of M’hiri et al. [25], were referred to the microwave treatment (10 s/170 W/3 cycles) to achieve enhancement of extraction, while ultrasonic treatment (30 min/135 W/3 cycles) came in the second order. Briefly, a successive extraction technique was applied to obtain the oil followed by the polar extract. The study used the microwave treatment of the byproduct materials (100 g/0.5 L petroleum ether (40–60); 10 s/170 W/40 °C) utilizing the equipment Sharp R-770AR ST Microwave at the same condition before, followed by the laboratory equipment of ultra-sonication (1 h/140 W, 40 °C), VCX 750 ΜLtrasonic probe, ΜLtrasonic Microprocessor, Thermo fisher scientific, SAS, France.

Aqueous ethanol was recommended here due to the high efficiency in minor components recovery [26]. Therefore, the polar extract was performed on the same samples that previously had been treated using the aqueous ethanol (80%) solvent by the same technique, instead of petroleum ether (40–60). Of the extracted oil, just a sample was taken for fatty acid composition analysis, and the remained oil was utilized for the mixture used in a liposome preparation.

### 2.3. Determination of the Volatile Contents

The experiments were carried out using an Agilent 7890A GC-apparatus with a split injector port set to 200 °C and a split ratio of 200:1. For static headspace sampling, an Agilent 7697A auto-sampler was employed. Before sampling with a 20μL loop (85 °C), vials were equilibrated at 75 °C for 8 min. Separation was achieved using a DB-624 capillary column with a continuous helium carrier flow of 1 mL/min. The initial heating rate for the oven was 45 °C for 3 min, accompanied by a ramp (3 min/240 °C) at a rate of 25 °C/min. The Agilent detector 5975 MSD was being used to identify and quantify essential compounds that distinguished including volatiles in the scan range (29–250 *m*/*z*; rate of 6.1 scans/s). The (B.09.00) Mass Hunter program was used to construct all calibration curves and sample concentrations.

### 2.4. Determination of Total Phenolic and Flavonoids 

A diluted solution of the Folin & Ciocalteus’ phenol reagent (1 mL) was combined with about 0.2 mL of extracted solution. After 3 min, sodium carbonate (0.8 mL/7.5%) was added, the mixture was allowed to rest for 30 min, and the absorbance was measured at 760 nm with a UV spectrophotometer (Shimadzu, Kyoto, Japan).In comparison to the blank, the total phenolic content (TPC) was measured and represented as mg gallic acid equivalent (GAE)/100 g sample. Using an aluminum chloride colorimetric technique [7], the total flavonoid content (TFC) of mandarin oil was measured. One milliliter of AlCl_3_ (2%, *w*/*w*) in methanol was combined with 1 mL aqueous methanol extract (1 mg/mL; 80% methanol) or normal catechol solution.

### 2.5. Determination of Antioxidant Activity

The antioxidant activity was determined for the prepared oil using two assays of the DPPH free radical scavenging assay according to the method of Shimada, et al. [27], with modifications described by Badr et al. [16]. The second method was the ABTS radical scavenging assay, which was referred to by Abdel-Fattah et al. [20]. The absorbance of the DPPH solution at 517 nm was measured using a Shimadzu spectrophotometer. In addition, the ABTS^+^ radical scavenging absorbance was taken at 734 nm using the same apparatus. All tests were carried out in triplicates. The inhibition of the radicals was calculated according to equation recommended before [19,20].

% of radicals inhibition = [(Abc − Abs)/(Abc)] × 100

where:

Abc = absorbance of the control solution.

Abs = absorbance of the test extract.

The previous equation was referred to calculate the DPPH radical scavenging and ABTS radical scavenging as described by Abdel-Fattah et al. [20].

### 2.6. Determination of Phenolic and Flavonoids Fractions

Stuper-Szablewska et al. [25] developed a procedure for measuring the bioactive fraction contents of the phenolic compounds in a previously produced extract of mandarin seeds. The phenolic content was determined at 320 and 280 nm by comparing retention times of analyte concentrations by a retention time for standards and injecting a certain proportion of the standard to the analyzed sample and conducting the experimenting. The detection limit was set at 10 ng/g material, and the findings were computed in triplicate and shown as mean ± SEM.

### 2.7. Estimation of Fatty Acids in Fixed Byproduct Oil

The oil content of fatty acid was determined according to the technique published by Abdel-Razek et al. [28]. To summarize, the Agilent 7890 apparatus (Agilent Technologies, Santa Clara, CA, USA) has been used to examine diluted oil, which was accompanied by the FID and capillary Innowax column (30 m × 0.20 mm × 0.20 mm). The carrier gas flow rate was 1.5 mL/min, and the column temperature was 210 °C. The results were provided as weight percentages after integrating and calculating the data using the Chem-Station and comparing the retention times to recognized standards.

### 2.8. Estimation of the Anti-Fusarium Activity for the Crude Extract, Trans-Ferulic, and Hesperidin

To determine the more effective application of phenolic compounds as individual applications and in their crude extract, two compounds (trans-ferulic and hesperidin) were chosen. These compounds were individually added to the fungal growth media at the same concentration that was determined in the crude extract. Briefly, five groups were designed for each fungal strain crude extract treatment, trans-ferulic treatment, hesperidin treatment, the control, and Nystatin (EIPICO chemical, Co., 10th of Ramadan City, Cairo, Egypt) as a standard antifungal [14]. The treatment materials were applied in the growth media of the five *Fusarium* strains identified in Section 2.1 of this study. The impacts were recorded as an inhibition zone (mm) using the agar diffusion assay as described before [16]. 

### 2.9. Preparation of the Extract as Liposome

The extracted materials were used for the preparation of the mixture that targeted liposome formation. Extracted materials of the byproducts were mixed using the emulsifier system between 1% and 80%. The resultant solution considered the mixture, which is utilized to form the liposome. The liposomal extract was prepared for use in the strategy for film technique, in which soybean phospholipids: cholesterol (5:1; *w*/*w*) [29] were prepared in a presence of between 1% and 20%, the resultant lipid phase was dissolved in dichloromethane. Then, this phase was mixed with the byproduct extract mixture, the two solutions were combined, and indeed the solvent was evaporated under vacuum using a rotary evaporator (45 °C/0.5 h), accompanied by drying in a hot air oven (60 °C/1 h).

### 2.10. Determination of the Liposomal Characterization 

Particle size, zeta potential (ζ), and the poly-dispersity index (PDI) were determined using the equipment of nano-ZS, Zeta-sizer, Malvern Instruments Ltd., Malvern, UK. The Ultrapure water was applied to dissolve the experimental samples at 1 mg/mL concentration then filtered through a filter-syringe (0.45-μm), which facilitated the removal of the insoluble particles.

### 2.11. Determination of Liposomal Formation Efficiency 

The efficiency of liposomal formation (ELF) was determined including a ratio between the extract concentrations in the solution used for the liposome preparation against the total applied quantity of extract used at the initial time. The ELF calculated as a percentage regarding equation recommended before [30] whichis described below:

% of the ELF = [(Inc − Dtc)/(Inc)] × 100

where:

Inc = extract applied concentration used for liposome formation.

Dtc = extract concentration in the solution after liposome formation.

The ELF values also were determined during the sample storage periods of 0, 5, 10, 20, 30 days for the evaluation of their stability. 

### 2.12. Determination of Antifungal Potency of Liposomal and Non-Liposomal Materials

The minimal fungicidal concentration was determined for extracts forms (liposomal and crude extract) using the methodology described by Badr et al. [31]. The antifungal potency of liposomal and non-liposomal materials was determined using the same methodology described before [32]. The fungal strains’ spore suspension (1.2 × 10^5^ spore/mL) of each was inoculated into conical flasks containing potato dextrose broth. Flasks, which were utilized for each fungal growth, are classified into three groups regarding the treatments (control, crude extract, and liposome). The results were described as the more mycelia weight reduction by the treatment the more antifungal potency material, where the reduction expressed including percentile ratio using the equation recommended by Badr et al. [21]:

% mycelia-weight reduction = [(Wc − Wt)/(Wc)] × 100

where:

Wc = weight of mycelia growth for the control.

Wt = weight of mycelia growth for treatment.

The hypha fungi of the liquid media were dried using a hot air oven using a pre-weighted filter paper until the weight became constant. The differences in hypha weight are considered in a correlation to the inhibition effect of the applied solution in media. The hypha weight of the control was the standard condition, while the treatment caused a reduction in hypha weight calculated compared to the control. 

### 2.13. Estimation of Extracted Materials on Mycotoxin Secretion 

The effect of applied materials of the crude and liposomal extract obtained from the byproducts was evaluated against toxigenic producing fungi in liquid media. In addition, parallel the effect of the individual applying for the standard materials of phenolic compound (trans-ferulic acid and hesperidin). The applied strains were *F. culmorum* KF846 as a producer of the ZEA and the DON toxins, while *P. verrucosum* NRRL965 was applied for ochratoxin A production. The estimation of extracted media was performed in the liquid growth media of each fungus including an individual treatment according to the methodology described before [18]. In brief, fifty milliliters of czapek-dox liquid media were prepared in 125 mL conical flasks. The flasks were inoculated by the fungi strains individually in groups; each group was classified regarding the treatments as the crude extract, liposomal extract, trans-ferulic acid, hesperidin, and the control. These treatments were investigated against the amounts that were secreted of the OCA, OCB, ZEA, and DON toxins compared to their regular secretion amount in the control flask.The flasks (control and treated flasks) were incubated for five days after the treated ones were supplemented by a concentration of targeted extract (250 μL/mL media), which was tested before including a minimal fungal concentration dose. The results of mycotoxin inhibition were expressed as ng/mL media and were performed in triplicates.

### 2.14. Determination of Mycotoxin Content 

The Agilent 1100 high-performance liquid chromatography, Agilent Technologies, Hewlett-Packard Strasse 876, 337 Waldbronn, Germany was used to determine mycotoxin in the studied media for secretion contents evaluation. For the separation and determination, an Extend-C18, Zorbax column (250 mm × 4.6 mm × 5µm, Agilent Co., Santa Clara, CA, USA) was utilized. The column temperature was held to 40 °C, and the flow rate was adjusted to 1.0 mL/min, with a sample and reference injection volume of 10 μL. For Zearalenone, the detector was configured to excite at 220 nm and emit at 330 nm, for ochratoxins, it was set to excite at 330 nm and emit at 450 nm. Deoxynivalenol was detected at 360 nm and 470 nm, for excitation and emission, respectively [33]. A Hewlett-Packard Chem-Station program Manager was being used to integrate and analyze data.

### 2.15. Statistical Evaluation

The findings were presented in means with standard error means (SEM) obtained from 3 replications. The data were statistically analyzed using ANOVA one-way test and the GraphPad Prism 7 was being applied to perform statistical analysis on data and graphics (GraphPad Software Inc., San Diego, CA, USA).

## 3. Results

It is important to evaluate the minor component contents of the extracted material. This will help the vision to illustrate the activity for both the crude and manufactured extract. Due to that, the study focuses on the next analyses to discover the hidden features of the extract.

### 3.1. Volatile Content Evaluation

Regarding the content of volatile component of the extracted byproduct materials, it was clear the majority content of Limonene component, where other volatiles such as α-Pinene, β-Pinene, β-Myrcene, β-phellandrene, γ-Terpinene, and α-Terpinolene are presented in considerable amounts. At the same time, two compounds were recorded to be not detected in the examined material of mandarin byproducts, which are known as Geranyl and α-Cubebene. Moreover, the compounds linalool, nonanal, and citronellal are recorded including traces by the GC-headspace analysis of the byproduct materials. It is worth mentioning that the group of the terpene compounds is present with a significant value, which may explain the future biological behavior of the extract.

### 3.2. Total Phenolic and Flavonoid Contents

Total phenolic contents of aqueous ethanolic solution give an impression of being much higher concerning the comparison between the regular and applied extraction system. The total phenolic content was recorded at 71.24 ± 5.48 mg/g DW, while the total flavonoid content was recorded at 94.68 ± 3.05 mg/g DW (Table 1). The data of the total phenolic content were created as derivatization of using a calibration curve of Gallic acid (0–500 μg/mL) and represented as gallic acid equivalents (GAE)/g DW, and the equation of the standard curve was y = 0.0016x − 0.915 (where R^2^ = 0.996). For the total flavonoid content, the results were performed including derivatization of practicing a calibration curve of catechol (0–500 μg/mL) and represented as catechol equivalents (Cat)/g DW and the equation of the standard curve was y = 0.0005x − 0.438 (where R^2^ = 0.998).

### 3.3. Antioxidant Activity for the Byproduct Extract 

**Figure 1:**
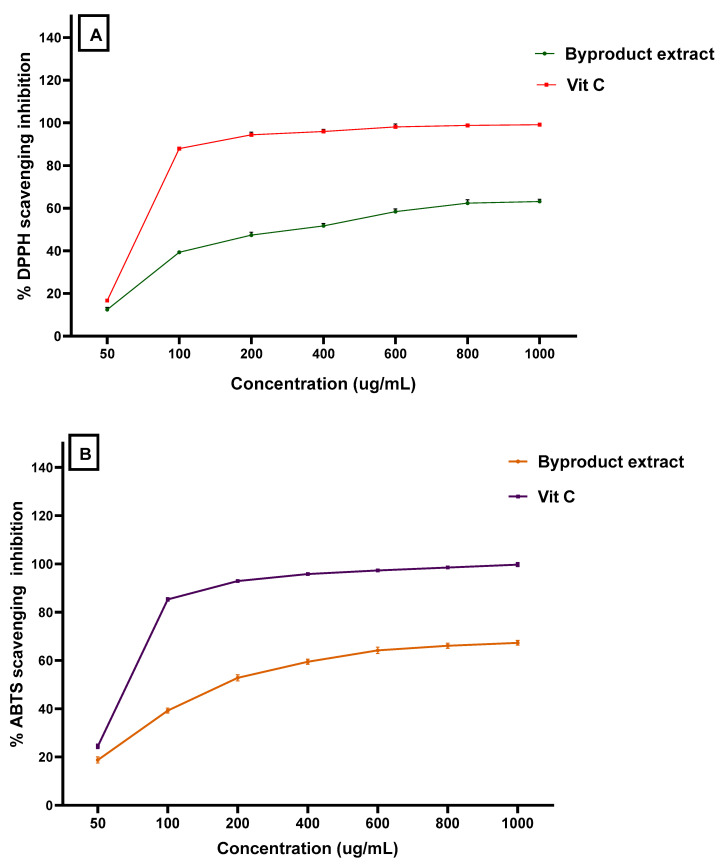
The antioxidant activity of mandarin byproduct using (**A**) DPPH assay, and (**B**) ABTS assay.

The total antioxidant capacity of the byproduct extract concerning the three assays reflects a moderate potency in comparison to ascorbic acid to be a standard antioxidant material (Figure 1). The antioxidant activity of the extract was shown by potency nearer to the values recorded by ascorbic acid that was applied as a standard antioxidant. This result was recorded by the DPPH assay as well as the ABTS + scavenging assay. In the same way, these assays were reflected by a valuable antioxidant activity. So, the results point out the expected potency of the byproduct extract wherever it could possibly be applied for its bioactivity in several.

### 3.4. Phenolic and Flavonoids Fractions Determination

The results, which are represented in Table 2, manifested the content of the extract regarding phenolic compounds. The results in Table 2 were classified into two columns for phenolic acids and flavonoids. Concerning the phenolic acids, the chlorogenic is followed by the protocatechuic and sinapic acids recorded by the major content of the extract. At the same time, p-hydroxybenzoic acid was determined by the lowest value among the phenolic acid contents. Moreover, the flavonoid content in the byproduct extract was shown by high contents of hesperidin, then kaempferol while the derivative of isorhamnetin-3-o-rutinoside came to be the third major compound. There were two compounds regarding the flavonoid compounds shown to be not detected, where the compounds were recognized as luteolin and chrysin. It is significant to point out the presence of catechol and quercetin with valuable amounts. 

### 3.5. Fatty Acids in Fixed Oil Byproduct

The content of the fatty acid composition, which was extracted from the byproduct materials using petroleum ether (40–60), was analyzed and the results were explored in Table 3. It was noticed that two fatty acids are recorded not detected, namely myristic acids and docosahexaenoic acids. The content of saturated fatty acid in this oil recorded not more than 16% of the total fatty acid composition. Moreover, the ratio between the percentage of the monounsaturated and the polyunsaturated fatty acids was shown to be close. In addition, the oil content was recorded by a variable content of omega fatty acids including ω3, ω5, ω6, ω7, and ω9, with a considerable content of ω6 close to 40% of the fatty acid composition. This majority of the ratio presenting may lead to a biological function by the oil application. However, myristoleic acid and palmitoleic acids represent the only content of ω5 and ω7 fatty acid content, respectively, and their ratios are shown at a considerable value.

### 3.6. Anti Fusarium Activity of the Crude Extract, Trans-Ferulic, and Hesperidin 

**Figure 2:**
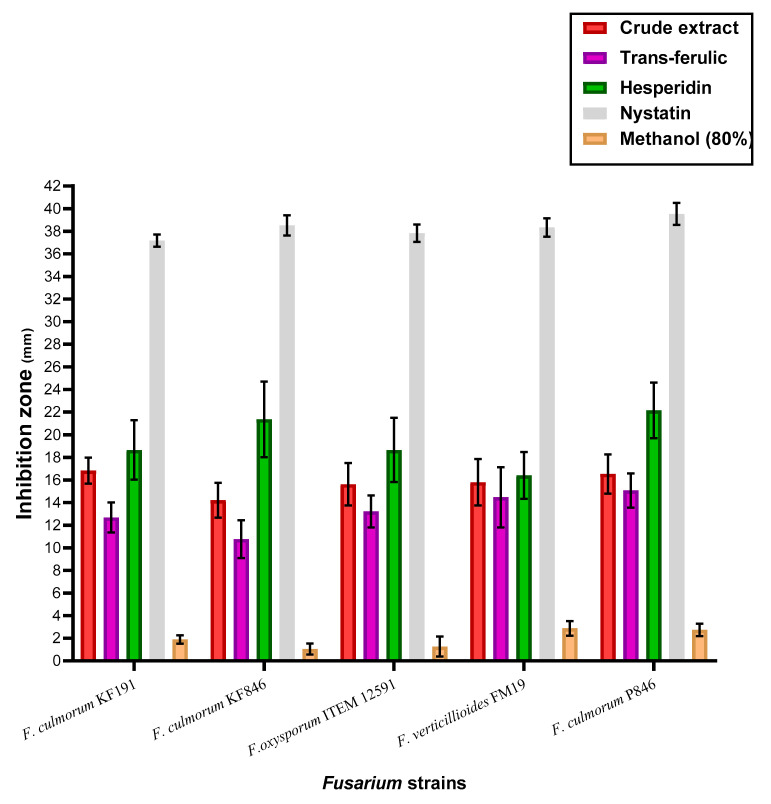
The anti-*Fusarium* activity of the crude extract, trans-ferulic, and hesperidin.

The estimation of antifungal activities for the trans-ferulic, hesperidin, and the byproduct crude extract was recorded to be not as being very apart (Figure 2). However, the inhibition effect of the applied treatments was shown by effective impact against all investigated strains of Fusarium fungi. The results against all applied types of fungal strains were recorded by complete inhibition using the positive standard Nystatin. In more specific terms, hesperidin, a standard flavonoid, was recorded as the most effective treatment leading to more growth-inhibition of the *Fusarium* strains. Again, the species of *F. culmorum* were recorded to be more influenced by the treatment, particularly the strains KF846 and P846. The strains of *F. culmorum* are fungal pathogens that cause seedling blight and other diseases in food-plant materials and have the capacity to produce mycotoxins. *F. culmorum* species are regarded as a single phylogenetic species, with no major linkage imbalance and little or no lineage development preceding the origin fungi of the genus *Fusarium*.

### 3.7. Liposomal Characterization Evaluation

Concerning the determination of the liposomal extract characterization, particle size dimensions were shown by an increment during the storage period. The changes in zeta potential values during 30 days of storage time were recorded by limited values. It was recorded at the initial time at −41.24 ± 2.08 mV and it was reach −31.05 ± 8.34 mV by the storage time ended. The values, which were recognized for the poly dispersing index shown as the highest value after seven days of the storage time, then decreased and stabilized until the end time of the storage; however, the liposomal system was shown still stable during the storage. Moreover, the efficiency of liposome formation during the storage period was recorded by decrement but still showed a good efficiency. 

### 3.8. Antifungal Potency of Crude and Liposomal Extracts

**Figure 3:**
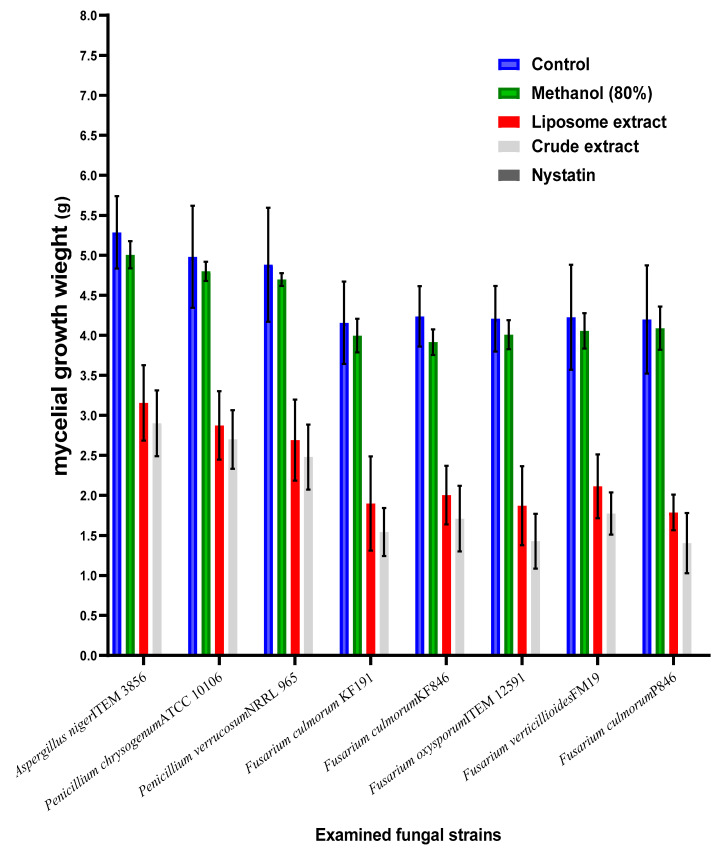
Antifungal effect of the crude and liposomal extracts of the byproducts.

The effect of crude byproduct extract and its liposomal form was evaluated against the control for inhibition effect of mycotoxigenic fungi (Figure 3). 

This inhibition was represented by mycelia growth losses after the treatment. Compared to the crude extract, the capsulated extract as liposome was more effective in fungal growth reduction. Once more, the strains related to Fusarium fungi, particularly *F. culmorum* species, were recorded as more sensitive to the extract component than other fungal strains. Moreover, the inhibition ratio for the growth of strains KF846 and P846 was recorded includingthe highest values, which points out to their high sensitivity to the extract influence. The impact of the two types of extracts (crude and liposome) also was effective against the strains of *A. niger*, *P. chrysogenum*, and *P. verrucosum* fungi. These strains were known to be ochratoxins-producing strains, where their presence in the raw or the manufactured material may lead to the occurrence of toxicity. The existence of natural components that possess fungal inhibition will organize the contamination level at the two levels (fungal, as well their metabolites). 

### 3.9. Estimation of the Impact on the Mycotoxin Secretion 

**Figure 4:**
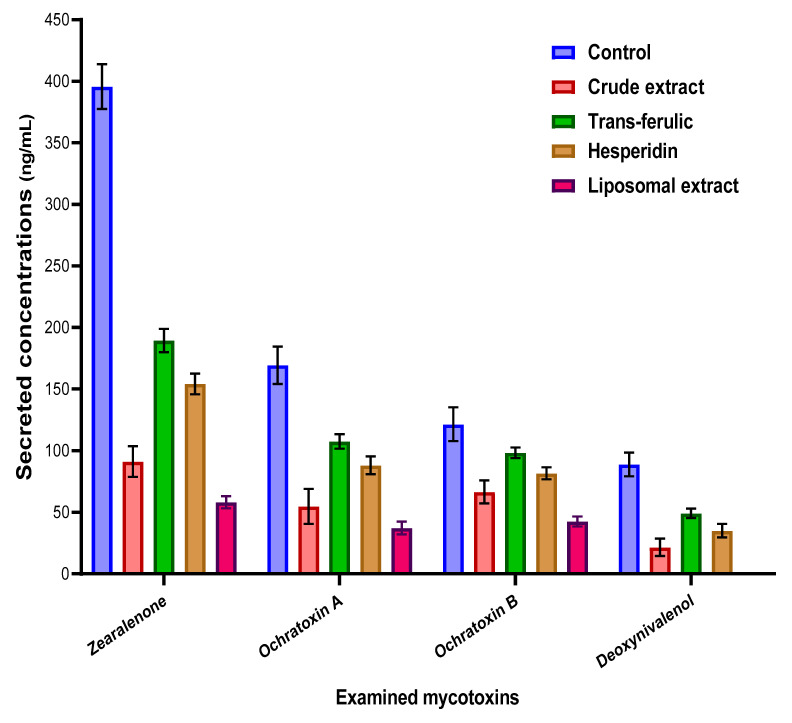
Anti-mycotoxigenic effect of the crude and liposomal extracts of the byproducts compared to two standards of the phenolic compounds.

The impact of crude byproduct extract, liposomal extract, and the two standard materials of phenolic compounds (trans-ferulic and hesperidin) was determined in the applied fungal growth media (Figure 4). The results reflected the efficacy of liposomal extract followed by the crude extract to reduce the secreted concentrations of mycotoxins. It was clear that the impacts of applied extracts (crude and liposomal extract) were recorded by more reduction to the ZEA concentrations followed by the DON concentrations. Nonetheless, the implementation of the fungal growth media of trans-ferulic and hesperidin was reflected as a valuable reduction in mycotoxin concentrations, but the reduction ratio was still less than the extract itself.

## 4. Discussion

The determination of bioactive molecules’ content was planned to evaluate the extracted materials impact on examined toxigenic fungi, and to help toward the illustration of their potency to reduce mycotoxin secretion in fungal growth media, regularly evaluating consideration concerning the components that possess antioxidant potency. Once more, the impact in comparison to the standard materials (trans-ferulic and hesperidin) was evaluated, as well as in comparison to the liposomal form of the extract. The extract contents of the minor components determined for the total extract consisted of fixed and volatile components. The content of the extract could refer to its expected bioactivity due to the majority of bioactive molecules. By the information about the molecule structure of the extract, the authors were able to explain the influence regarding the toxigenic fungi and their metabolic products, mycotoxins.

Phenolic content determination is the process of determining a phenolic quantity in samples. Phenolic compounds comprised in the phytochemical extract possess redox features, which corroborative their action being antioxidants [41]. The mention of these contents supports the theory of oxidative stress reduction by their existence in the media. Phytoconstituent contents of the extract possess hydroxyl groups that help increase the properties to act as free radical scavenging agents [42,43]. Concerning this, the results obtained to be fungal inhibition for both the growth on solid media (Figure 2) and the growth on liquid media (Figure 3) are connected partially to the extract contents of phenolic acids that existed in the extract (Table 2). These acids are included by trans-ferulic, chlorogenic, protocatechuic, and sinapic acids that are recorded to be the major content of the phenolic acids. While, trans-ferulic [44], chlorogenic [45], protocatechuic [46] are reported to have antifungal effects, the phenolic acid of sinapic was reported by antifungal and antimycotoxigenic effect [47]. Moreover, the flavonoids, which are members of phenolic compounds, were shown present in the extract in significant amounts. The dominant content was ordered in descending as hesperidin, kaempferol, catechol, and quercetin that also were known to possess antifungal activity [48,49].

Nevertheless, the extract contents of phenolic compounds, particularly flavonoids, supported their antioxidant activities (Figure 1). The antioxidant properties are related to the hydroxyl groups that are known to be rich in flavonoid compounds [50]. The antioxidant activity related to the hydroxyl group increases when its position was connected to the aromatic compound [51]. This gives the extract a feature to act as an antimicrobic agent where it has existed. The extracted content of the essential components was shown by concentrations regarding molecules that provide antimicrobial activity by their existence. These molecules include limonene, γ-terpinene, α-pinene, β-pinene, β-myrcene, and α-terpinolene. The existence of such components also participates in the explanation of the high antifungal impact of the extract. 

The third factor, which supports the antimicrobial and antifungal effects of the crude byproduct extract, is the implementation of a minor amount of fixed oil. The composition of this oil concerning the fatty acids content was recorded in Table 3, in which some fatty acids with known antifungal activity were presented. It is important to refer that, oleic, linoleic, and palmitoleic acids have existed in considerable content, forming the majority of fatty acid content. Again, the considerable content of omega fatty acids that are manifested in variable types (Table 3), strongly points out the expected antifungal activity function occurred by the application of this extract in microbial media [38].

In this regard, the anti-*Fusarium* effect of the extract was determined in comparison to the standard phenolic compounds of trans-ferulic acid and hesperidin (Figure 2). By excluding the result of Nystatin standard antifungal, the hesperidin effect as anti-*Fusarium* was recorded by the most effective treatment, followed by the crude extract. Hesperidin’s impact, similar to an anti-*Fusarium* treatment, could be related to its high content of hydroxyl-aromatic groups, which are known by antifungal effect [50,51]. While the crude extract still possesses a high effect as anti-*Fusarium*, and this impact is linked to the minor components that have antioxidant activity. This gives an idea for saving this impact in a more suitable form such as the liposomal form. The results of liposome characteristics reflect stability for the formed system (Table 4). However, the efficiency during the storage period recorded by significant values may take into consideration including a moderate release system recommended for the crops-coating application. 

To evaluate the importance of crude extract saving by liposome application, it was tested compared to the crude extract for the inhibition effect of several toxigenic fungal strains (Figure 3). The effective impact between the application of the crude and the liposome form was recorded by narrow-scale changes but is still better than the crude extract compared to the control. In addition, the effect was clear against the strains of *Aspergillus, Penicillium,* and *Fusarium* fungi. The next point was connected to the evaluation of the applied materials on mycotoxin secretion in the liquid media of fungal growth. Four mycotoxins were targeted to evaluate: namely OCA, OCB, DON, and ZEA. Ochratoxins were targeted to determine using the media growth of *P. verrucosum*, while DON and ZEA are targeted to estimate in *F. culmorum* fungi. The results were reflected by the high efficiency of both the crude and liposome forms of the extract compared to the control. 

**Figure 5:**
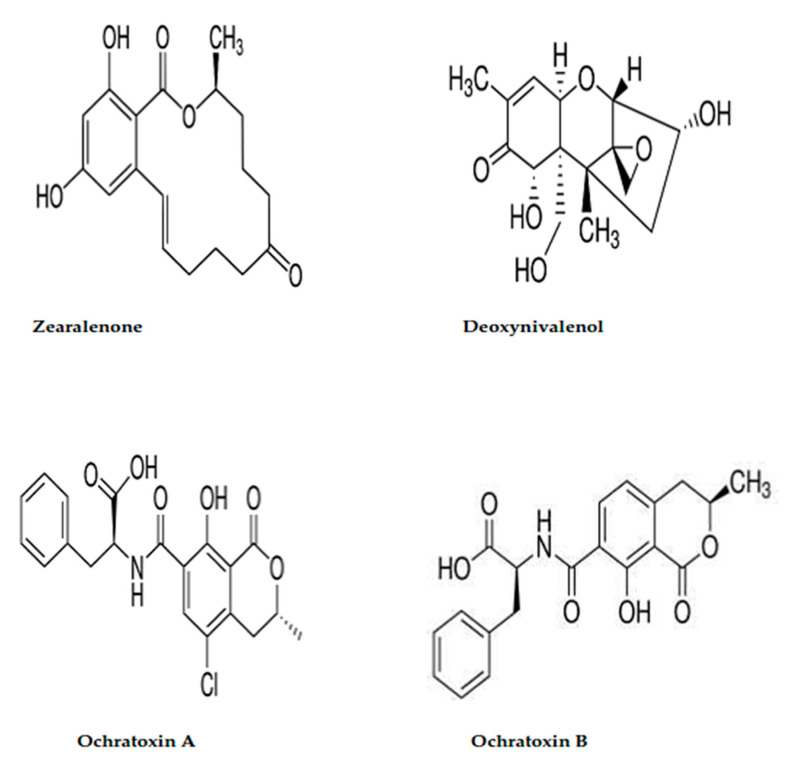
Chemical structure for ochratoxins (**A**,**B**), deoxynivalenol, and zearalenone.

Due to the chemical structure of the applied mycotoxins that was shown in Figure 5, hydroxyl, ketone, and amid groups have existed to be the active groups. The molecules of ochratoxins were distinguished by the presence of the amide group, as well as two hydroxyl groups. The structure of zearalenone is distinguished by the existence of lactone ring and dihydroxybenzene derivative. About the DON, it is featured by the presence of 7 stereocenters in the molecules. These centers played a significant function in the transformation between the molecules isomers when interacting in reactions [52]. This suggestion could explain the more activity of applied materials to reduce the DON concentration, and particularly the liposome formed by the crude extract. The recognized content of the active groups of secreted toxins, aside from the activity of extract content of bioactive molecules that inhibit the fungal growth rate, leads finally to the recording of mycotoxin degradation.

## 5. Conclusions

*Fusarium* and its toxins are considered a significant hazard for food. The novel strategies to reduce this contamination are becoming urgent. The extract of mandarin byproduct (peels have short stalks with small leaves) as anti-*Fusarium* was evaluated. The results indicate valuable extract contents of phenolic acid, flavonoids, limonene, pinene, and terpenes. Crude extract reflects a significant effect compared to the standard material possessing anti-*Fusarium* fungal growth, which gives a piece of evidence for the expected impact to minimize mycotoxins. The synergistic effect between the minor components included in the extract leads to the anti-*Fusarium* influence. The extract was transformed into the liposome to save this influence and was applied to examine against the standard compounds (trans-ferulic, hesperidin) for their anti-mycotoxigenic impact. The results recommended the liposome, followed by the crude extract, as the best reduction in mycotoxins. The reduction could be illustrated through the effect on the growth rate of fungi. The variation of mycotoxins’ chemical structure related to applied fungi may explain the differentiation recorded for the extract effect. This study opened a research gateway toward the impact of natural extracts utilized as a neoteric approach in mycotoxin reduction.

## Figures and Tables

**Table 1:** Volatile compounds content determined in mandarin byproducts extract.

Compound	RI	Byproduct Content	Identification
Hexanal	801	0.37 ± 0.05	MS and RI
α-Thujene	928	2.67 ± 0.31	MS and RI
α-Pinene	939	3.41 ± 0.54	MS, RI and ST
Sabinene	972	0.88 ± 0.12	MS and RI
β-Pinene	981	4.57 ± 0.24	MS, RI and ST
β-Myrcene	991	7.19 ± 0.81	MS and RI
Octanal	1006	0.39 ± 0.14	MS and RI
α-Terpinene	1012	0.84 ± 0.16	MS, RI and ST
β-phellandrene	1030	3.12 ± 0.06	MS and RI
Limonene	1033	67.54 ± 1.74	MS and RI
γ-Terpinene	1074	4.82 ± 0.98	MS, RI and ST
α-Terpinolene	1096	2.36 ± 0.49	MS and RI
Linalool	1100	0.08 ± 0.03	MS, RI and ST
Nonanal	1104	0.02 ± 0.001	MS and RI
Geranyl	1149	ND	MS and RI
Citronellal	1159	0.03 ± 0.002	MS, RI and ST
Decanal	1234	0.14 ± 0.01	MS and RI
Ethanone	1274	0.27 ± 0.05	MS and RI
Cadinene	1275	0.34 ± 0.08	MS, RI and ST
α-Cubebene	1345	ND	MS and RI
Isopiperitone	1473	0.18 ± 0.02	MS and RI
α-Sinensal	1526	0.15 ± 0.03	MS and RI
β-Sinensal	1675	0.54 ± 0.14	MS and RI

The results are represented includingmeans ± SEM, where (*n* = 3). ND: Not Determined.

**Table 2:** Chemical constituents of phenolic compound contents of mandarin byproduct extract.

Phenolic Acids	Concentrations (mg/100 g)	Flavonoids Compounds	Concentrations (mg/100 g)
Gallic acid	51.77 ± 2.84	Catechin	84.52 ± 1.08
Chlorogenic acid	125.13 ± 1.05	Catechol	125.24 ± 2.74
Protocatechuic acid	122.31 ± 1.94	Epicatechins	27.41 ± 0.88
*trans*-Ferulic acid	91.74 ± 1.93	Rutintrihydrate	35.17 ± 1.14
*trans*-Cinnamic acid	25.22 ± 1.05	Apigenin 7 glucoside	44.27 ± 1.67
Vanilic acid	22.7 ± 0.87	Quercetin	63.08 ± 4.51
Caffeic acid	34.58 ± 1.41	Luteolin	ND
Ferulic acid	235.54 ± 3.34	Hesperidin	492.11 ± 1.15
*p*-Hydroxybenzoic acid	2.94 ± 0.67	Naringenin-7-o-glucoside	13.97 ± 0.54
*p*-Coumaric acid	60.54 ± 1.08	Kaempferol	268.56 ± 3.54
Syringic acid	19.91 ± 1.18	Isorhamnetin-3-o-rutinoside	152.81 ± 2.78
Sinapic acid	121.75 ± 2.86	Chrysin	ND
Total Phenolic acids	914.36 ± 20.22	Total Flavonoids	1307.14 ± 20.03

The results are represented including means ± SEM, where (*n* =3).

**Table 3:** Fatty acid composition of the oil extracted from mandarin byproducts.

Carbonnumber	Fatty Acids	Concentration (%)	Notes	Reference for Activity Impact
C 12:0	Lauric	0.88 ± 0.21	Short-chain	-
C 14:0	Mayristic	ND	Not detected	-
C 16:0	Palmitic	0.84 ± 0.11	Less than 1%	-
C 18:0	Stearic	4.22 ± 0.41	Antifungal impact	[34]
C 20:0	Arachidic	10.09 ± 0.22	Antifungal impact	[35,36]
C 22:0	Behenic	0.48 ± 0.08	Less than 1%	-
C 24:0	Lignoseric	0.16 ± 0.04	Less than 1%	-
**Omega Fatty acid contents**				
C 18:3 **n-3**	Linolenic	8.72 ± 0.37	Antifungal impact	[37]
C 20:5 **n-3**	Ecosapentanoic	0.09 ± 0.005	Antimicrobial impact	[38]
C 22:6 **n-3**	Docosahexaenoic	ND	Not detected	-
C 14:1 **n-5**	Myristoleic	10.61 ± 0.54	High content	
C 18:1 **n-9**	Oleic	14.71 ± 0.88	Antifungal impact	[38]
C 18:2 **n-6**	Linoleic	18.44 ± 1.05	Antifungal impact	[38]
C 20:4 **n-6**	Arachidonic	0.82 ± 0.04	Less than 1%	-
C 20:2 **n-6**	Eicosadienoic	0.05 ± 0.003	Antimicrobial impact	[39]
C 22:2 **n-6**	Docosadienoic	7.19 ± 0.83	Antimicrobial impact	[40]
C 16:1 **n-7**	Palmitoleic	21.74 ± 0.63	Major content	
C 20:1 **n-9**	Gadoleic	0.26 ± 0.01	Antimicrobial impact	[36]
C 20:3 **n-9**	Eicosatrienoic	0.63 ± 0.08	Less than 1%	-
C 22:1 **n-9**	Erucic	0.02 ± 0.001	Trace content	-
C 24:1 **n-9**	Nervonic	0.05 ± 0.001	Trace content	-
	SFA/MUFA/PUFA	0.25:1.42:1.33		
	Cox value			

The results are represented as means ± SEM, where (*n* = 3); ND represented the not detected values. (**n-**): referred to the omega fatty acids where numbers referred to their type.

**Table 4:** The changes of particle size, zeta potential, poly dispersing index, and liposome formation efficiency during the storage.

Storage Period (Days)	Particle Size (nm)	Zeta Potential (mv)	PDI	LFE
1	89.41 ± 2.11	−41.24 ± 2.08	0.284 ± 0.002	99.21%
3	96.37 ± 4.27	−40.7 ± 3.61	0.373 ± 0.005	98.04%
7	107.66 ± 5.44	−38.14 ± 4.27	0.561 ± 0.004	91.18%
14	145.81. ± 4.81	−34.81 ± 4.56	0.418 ± 0.002	87.24%
21	177.64 ± 5.74	−33.63 ± 5.41	0.420 ± 0.008	82.15%
30	237.36 ± 8.63	−31.05 ± 8.34	0.416 ± 0.005	77.54%

The results are represented as means ± SEM, where (*n* = 3). PDI: poly dispersing index; LFE: liposome formation efficiency.

## Data Availability

The data in this study are available in this article.
